# Network meta-analysis on the log-hazard scale, combining count and hazard ratio statistics accounting for multi-arm trials: A tutorial

**DOI:** 10.1186/1471-2288-10-54

**Published:** 2010-06-10

**Authors:** Beth S Woods, Neil Hawkins, David A Scott

**Affiliations:** 1 Oxford Outcomes Ltd., Oxford, UK; 2 University of York, York, UK

## Abstract

**Background:**

Data on survival endpoints are usually summarised using either hazard ratio, cumulative number of events, or median survival statistics. Network meta-analysis, an extension of traditional pairwise meta-analysis, is typically based on a single statistic. In this case, studies which do not report the chosen statistic are excluded from the analysis which may introduce bias.

**Methods:**

In this paper we present a tutorial illustrating how network meta-analyses of survival endpoints can combine count and hazard ratio statistics in a single analysis on the hazard ratio scale. We also describe methods for accounting for the correlations in relative treatment effects (such as hazard ratios) that arise in trials with more than two arms. Combination of count and hazard ratio data in a single analysis is achieved by estimating the cumulative hazard for each trial arm reporting count data. Correlation in relative treatment effects in multi-arm trials is preserved by converting the relative treatment effect estimates (the hazard ratios) to arm-specific outcomes (hazards).

**Results:**

A worked example of an analysis of mortality data in chronic obstructive pulmonary disease (COPD) is used to illustrate the methods. The data set and WinBUGS code for fixed and random effects models are provided.

**Conclusions:**

By incorporating all data presentations in a single analysis, we avoid the potential selection bias associated with conducting an analysis for a single statistic and the potential difficulties of interpretation, misleading results and loss of available treatment comparisons associated with conducting separate analyses for different summary statistics.

## Background

Network meta-analyses enable us to combine trials that compare different sets of treatments, and form a network of evidence, within a single analysis [1] and to use all available direct and indirect evidence to inform a given comparison between treatments. Network meta-analysis is based on the assumption that, on a suitable scale, we can add and subtract within-trial estimates of relative treatment effects i.e. the difference in effect between treatments A & B (*d*_*AB*_) is equal to the difference in effects between treatments A & C and B & C (*d*_*AB *_= *d*_*AC *_- *d*_*BC*_) [1-3].

In this paper we show how network meta-analyses of survival endpoints can be conducted on the hazard ratio scale when some or all trials report cumulative count data. We also describe how trials with more than two arms reporting relative treatment effects (such as hazard ratios) should be included.

A survival endpoint is one where, over time, an increasing number of patients experience an event. Although death is the ultimate survival endpoint, many other endpoints may also be considered as survival endpoints. For example, in the study of epilepsy, seizure freedom may be regarded as a survival endpoint as over time the number of patients experiencing one or more seizures can only increase and the probability of being seizure free decreases. In contrast, the number of patients with a greater than 50% reduction in seizure frequency relative to baseline is not a survival endpoint as the number of patients achieving this response can increase or decrease over time.

Data on survival endpoints may be expressed either in the form of hazard ratio statistics derived from parametric or non-parametric methods of survival analysis [4,5] or as cumulative count statistics (the total number of subjects who have experienced an event at a specific time point). Unlike cumulative count statistics, hazard ratio statistics from survival analysis account for censoring, incorporate time to event information, and may be adjusted for co-variables. Where both hazard ratio and cumulative count statistics are available for a given study, use of the hazard ratio data may therefore be considered preferable.

The methods described in this paper allow hazard ratio and cumulative count survival statistics to be combined within a single network meta-analysis on the log-hazard scale; this might be termed a multi-statistic evidence synthesis. Treatment effects can then be estimated based on an inclusive set of data, and separate analyses for hazard ratio and count statistics are avoided [6-9].

Network meta-analyses should account for the correlations in relative treatment effect estimates that arise from trials with more than two treatment arms (multi-arm trials) [10]. These correlations are accounted for 'by default' when count statistics for individual trial arms are included in a network meta-analysis [10]. When hazard ratio statistics are used, we show how these correlations can be accounted for by deriving estimates of the mean log hazards (and their variances) for individual trial arms.

## Methods

We use an example data set describing mortality in randomised controlled trials of treatments for chronic obstructive pulmonary disease (COPD). The data set consists of a subset of trials from Baker et al [11]. We chose five trials providing mortality data for the following comparators: salmeterol, fluticasone, salmeterol fluticasone combination (SFC) or placebo. Three of the trials report count data [12-14], one reports hazard ratio data from a two arm trial [15] and one reports hazard ratio data from a multi (four)-arm trial [16]. Hazard ratio data were obtained from the trial publications and an existing meta-analysis of hazard ratio data [17].

The count statistics used are presented as Table 1 and the hazard ratio statistics as Table 2. The derived estimates of the mean log hazard ratio and it's standard error, required for the analysis, are also presented in Table 2, these were estimated using formulae (1) and (2):(1)(2)

**Table 1:** Count Statistics

Author/Trial (Date)	Treatment	r (deaths)	N (patients)
Boyd (1997) [12]	Salmeterol	1	229
	Placebo	1	227
Calverly/TRISTAN (2003) [13]	Fluticasone	4	374
	Salmeterol	3	372
	SFC	2	358
	Placebo	7	361
Celli (2003) [14]	Salmeterol	1	554
	Placebo	2	270

The results of this example analysis are purely illustrative of the methodology and do not provide any indication of the comparative effectiveness of treatments for decision-making purposes, as the data set omits relevant direct and indirect data.

Using the example data set we show how to:

(a) Perform a meta-analysis of count statistics on the log hazard ratio scale;

(b) Reflect correlation in relative treatment effect estimates from multi-arm trials;

(c) Combine count and hazard ratio statistics in a single analysis on the log-hazard ratio scale; and

(d) Include a random effect in to the analysis, whilst preserving the correlation in relative treatment effects for multi-arm trials.

We also discuss how other possible presentations of survival data, not available from our motivating example, could be incorporated into an analysis on the log-hazard ratio scale.

All analyses described were conducted using WinBUGS [18]. The WinBUGS code for the fixed and random effects analyses are presented as an Appendix.

(a) Meta-analysing count statistics on the log hazard ratio scale

The count data are incorporated in the network meta-analysis model using a binomial likelihood:(3)

where *r*_*s,k *_is the cumulative count of subjects who have experienced an event in arm *k *of study *s*; *n*_*s,k *_is the total number of subjects in arm *k *of study *s*; and *F*_*s,k *_is the cumulative probability of a subject having experienced an event (or 'failure').

A log cumulative hazard for each trial arm ln(*H*_*s,k*_) is then derived from *F*_*s,k*_.(4)

The log cumulative hazard estimates are then included in a treatment effect model with a linear regression structure. The log cumulative hazard is estimated as the sum of a study specific 'baseline' term *α*_*s *_and a treatment effect coefficient *β*_*k*_:(5)

where *β*_1 _= 0 for the reference treatment (placebo in our example) and *β*_*b *_represents the treatment effect for the baseline treatment in study *s*. The fixed study level 'baseline' term is a nuisance parameter, included to ensure that the treatment effect estimates are informed by within trial differences between treatment arms and not by differences in baseline event rates across trials.

Under an assumption of proportional hazards, the *β*_*k *_coefficient is equal to both the log cumulative hazard ratio and the log hazard ratio:(6)

where *h*_*s,b *_represents the hazard for the baseline treatment in study *s*. This identity allows us to combine the count statistics analysed on the log cumulative hazard scale with the hazard ratio data analysed on the log hazard scale. It also demonstrates that analysis of count statistics on the log hazard scale does not require a stronger assumption than proportional hazards.

(b) Reflecting correlations in relative treatment effects from multi-arm trials

Estimates of relative treatment effects from trials with more than two treatment arms will be correlated [10]. In our example the TORCH trial [16] is the only multi-arm trial reporting hazard ratio data. Estimated treatment effects from TORCH will be correlated, for example, the hazard ratio comparing SFC to placebo and the hazard ratio comparing salmeterol to placebo will be correlated due to their joint dependence on the time to event data in the placebo arm.

If a network meta-analysis is based on estimates of treatment effect in individual trial arms rather than estimates of relative treatment effect between arms ("contrast" statistics), this correlation will automatically be captured in the analysis [10]. This is the case when a network meta-analysis is based on count statistics. However, if the network meta-analysis is conducted based on estimates of relative treatment effect, this correlation between arms will not automatically be captured [10].

For multi-arm trials reporting hazard ratio statistics, this problem can be addressed by converting the log hazard ratios (contrast statistics) to log hazards (arm-specific statistics). Log hazards for individual trial arms are derived by nominally setting the log hazard for the baseline treatment *b*for the trial to zero. The mean log hazards for the other treatments are then equal to the log hazard ratios compared to baseline treatment.

The variance for a log hazard ratio is the sum of the variances for the individual log hazards. Standard errors of the log hazards for each trial arm can therefore be estimated by solving simultaneous equations based on the standard errors for the set of log-hazard ratios. For example:(7)

Where *se*^2^_*i,j *_is the variance of the log hazard ratio comparing arm *i *to arm *j *and *se*_*i *_is the standard error of the log hazard for arm *i*.

The standard errors of the log hazards for the other treatment arms are then estimated as:(8)

Mean log hazards and associated standard errors for each treatment in TORCH [16] are reported in Table 3. These are derived from the hazard ratio data presented in Table 2.

**Table 2:** Hazard ratio and log hazard ratio statistics

Author/Trial (Date)	Treatment	Base	HR	**HR**_**LCI**_	**HR**_**UCI**_		
Burge/ISOLDE (2000) [15]	Fluticasone	Placebo	0.76	0.51	1.13	-0.276	0.203
Calverly/TORCH (2007) [16]	SFC	Placebo	0.811	0.670	0.982	-0.209	0.098
	Salmeterol	Placebo	0.857	0.710	1.035	-0.154	0.096
	Fluticasone	Placebo	1.056	0.883	1.264	0.055	0.092
	SFC	Salmeterol	0.946	0.777	1.151	-0.056	0.100
	SFC	Fluticasone	0.768	0.636	0.927	-0.264	0.096

**Table 3:** Log hazard statistics for a multi-arm trial (TORCH [16])

Comparator	Log-hazard	se(log-hazard)
SFC	-0.209	0.072
Salmeterol	-0.154	0.070
Fluticasone	0.055	0.063
Placebo	0.000	0.066

In order to estimate standard errors of the log hazards for each treatment, we required estimates of the uncertainty associated with four treatment contrasts. In some cases this data may not be available and thus the methods presented in equations 7 and 8 may not be feasible. For example, hazard ratios and associated measures of uncertainty may only be available for each active treatment relative to a single common comparator (e.g. placebo) as is commonly reported in the published literature.

In this situation, we can approximate the standard error for the comparison between active treatments by assuming the standard error is proportional to . For example(9)

(c) Combining count and hazard ratio statistics in a network meta-analysis

The log hazard ratio statistics from two arm trials comparing treatments *k* to *b* are incorporated in the network meta-analysis model using a normal likelihood:(10)

where  is the log hazard ratio estimate for study s comparing treatments *k *to *b *and  is the corresponding variance.

The log hazard ratio estimates are then included in a treatment effect model with a linear regression structure, with the predicted log hazard ratio for a study *s *comparing treatments *k *and *b *equal to the difference between the two treatment coefficients:(11)

where *β*_1 _= 0 for the reference treatment (in our example placebo) and *β*_*b *_represents the treatment effect for the baseline treatment in study *s*. As in equation 5, the *β*_*k *_coefficient is equal to the log hazard ratio for treatment *k *compared to the reference treatment.

The log hazard statistics from a multi-arm trial are incorporated in the analysis using the following likelihood functions. For the baseline treatment, *b*:(12)

For the other treatments:(13)

where  is the log hazard for treatment arm *k *from study *s *and  is the associated variance.

The log hazard estimates are then included in a treatment effect model with a linear regression structure. The log hazard is estimated as the sum of a study specific 'baseline' term *α*_*s *_and a treatment effect coefficient *β*_*k*_:(14)

where *β*_1 _= 0 for the reference treatment (placebo in our example) and *β*_*b *_represents the treatment effect for the baseline treatment in study *s*. The fixed study level 'baseline' term is a nuisance parameter, included to ensure that the treatment effect estimates are informed by within trial differences between treatment arms and not by differences in baseline event rates across trials. As the *β*_*k *_coefficient is equal to the log hazard ratio for the cumulative count data, the log hazard ratio data and the log hazard data, they can be combined within a single analysis. Where an individual study reports both cumulative count and hazard ratio data, only one set of data should be included in the analysis to avoid double counting.

(d) Incorporating a random effect

In a random effects analysis of a network containing multi-arm trial contrast data, the correlation in the random effects must also be taken in to account. Again this is due to the joint dependence of the multiple contrast estimates on common trial arms.

This correlation is reflected in the model by separating the random effect deviation for each contrast in to the contributions to the random effect deviation of the two treatments that form the contrast. This is achieved by modifying the linear predictor component of the model for the cumulative count, log hazard ratio and log hazard data:(15)(16)(17)

where *re*_*s,k *_is the random effect deviation for arm *k *of study *s *and is assumed to be normally distributed with zero mean and variance *σ*^2 ^/2 where *σ*^2 ^is the random effect variance for a treatment contrast:(18)

This approach assumes that *σ*^2 ^is the same for all treatments and consequently that the random effect variance will be the same for all treatment contrasts. The assumption of a common random effect variance across treatment contrasts implies that the covariance for any pair of treatment contrasts from the same study will equal half the treatment contrast random effect variance [10].

A vague prior for the study specific baseline *α*_s_~*N*(0,10^6^) is used to ensure estimates of treatment effect are informed by within trial differences between treatment arms, and not by differences in absolute response between trials. A vague prior is also used for the treatment effect coefficients with *β*_*k *_~ *N*(0,10^6^) and *β*_1 _= 0 (representing placebo).

Each model was run for 40,000 burn-in simulations and 200,000 runs which were then thinned every 20^th ^simulation to reduce autocorrelation.

Two sets of initial values were used and convergence was assessed by examining caterpillar plots and Brooks Gelman-Rubin (BGR) statistics. The deviance information criteria (DIC) was used to compare the fit of the fixed and random effects models [19].

## Results

Results of the fixed and random effects models are presented as Table 4. The results provide no evidence that the random effects model is preferred; the DIC for the random effects model is marginally higher (lower DIC values are preferred, with differences of 2-5 considered important [19,20]) and the high level of uncertainty around the random effects standard deviation estimate indicates that there is little information to inform the random effect parameter.

**Table 4:** Network meta-analysis results

Comparator	Hazard ratio (95% CI) vs. placebo - fixed effects	Hazard ratio (95% CI) vs. placebo - random effects
Fluticasone	0.99 (0.84, 1.16)	0.89 (0.39, 1.42)
Salmeterol	0.82 (0.68, 0.98)	0.73 (0.29, 1.23)
SFC	0.78 (0.64, 0.93)	0.69 (0.26, 1.21)
random effect SD	-	0.36 (0.31)
DIC	25.25	25.73

**Figure 1:**
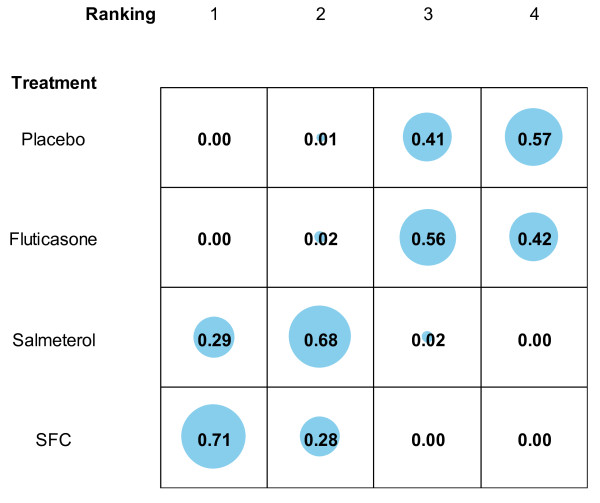
**Probability of treatments rankings**.

Figure 1 provides a presentation of the uncertainty in the analysis, showing the probability that each treatment takes each possible ranking (1st best, 2nd best, etc). For example, the figure tells us that there is a very low probability that fluticasone is the first or second most efficacious treatment in this analysis and that there is a 56% probability that is the third best and a 42% probability that it is the worst treatment.

Again it must be noted that this example data set is not an appropriate basis for answering the clinical question of which is the most efficacious treatment with respect to the mortality endpoint.

## Discussion

In this tutorial we have described how network meta-analysis can be conducted on the log-hazard ratio scale when the data on the survival endpoint available from the network of studies takes varying forms: count statistics and hazard ratio statistics from two and multi-arm trials.

In highlighting the importance of network meta-analysis as an extension to conventional pairwise meta-analysis Ades et al point out that "...to ignore indirect evidence either makes the unwarranted claim that it is irrelevant, or breaks the established precept of systematic review that synthesis should embrace all available evidence" [6]. A similar criticism can be levelled at meta-analyses that omit data points on the basis of the summary measure reported. This may result in intentional or unintentional selection bias. Furthermore, even if separate analyses are conducted for each summary measure available, this may have undesirable consequences by: producing conflicting results; producing coherent results that would not be supported by an 'all embracing' analysis; and unlinking some comparators from the network entirely.

Other authors have presented methods for combining arm-based mean change from baseline data with contrast mean difference data [21] and for combining mean time, median time and count data within a parametric survival model [22]. Further research is required in this area, for example in to methods for combining incidence rate data with count data, as these summary measures are often reported interchangeably across trials.

Other approaches to combining alternative reporting of time to event data have been discussed [8,23-26], recommended [27] and implemented [28,29]. These approaches involve the approximation of the log hazard ratio and its variance using available count statistics. The methods used in this tutorial avoid the use of approximations, instead utilising cumulative count statistics directly in the analysis.

A further common statistic reported for survival endpoints is arm-specific median survival time. This data can be incorporated using a similar approach as for count data. A binomial likelihood is used to incorporate the number of subjects experiencing an event (half the number of patients) and the number of patients analysed in to the model:(19)

Note that *r*_*s,k *_should be calculated outside the model to ensure it is a whole number. A log hazard for each trial arm ln (*h*_*s,k*_) is then derived from the cumulative failure probability *F*_*s,k *_and median survival time *T*_*s,k*_:(20)

The arm-specific log hazard estimates are then included in a treatment effect model that takes the same form as that for hazard ratio reporting multi-arm trials (see equation 14). It should be noted that analysis of median survival times requires the strong assumption of a constant hazard in each trial arm. The code for incorporating median survival time data is also provided in the Appendix.

Where multiple statistics are reported for a trial, hazard ratio statistics should be used in preference to median survival time and count data as hazard ratio statistics incorporate information about time to event and censoring, and analysis of hazard ratio statistics does not require assumptions stronger than proportional hazards. If both median survival time and count data are reported, a judgement is required to weigh up the relative merits of each presentation, as median survival time incorporates information about censoring but its analysis requires the stronger assumption of constant hazards.

Finally, this tutorial discusses methods for running analyses on the log hazard ratio scale. However, the question of which is the most appropriate scale for a given analysis (the scale on which transitivity and exchangeability are most likely to hold) - is an empirical question. Further work is required to develop methods for selecting the most appropriate scale for a given data set.

## Conclusions

Meta-analysis of summary statistics continues to play an important role in medical research [30,31]. Where only summary statistics are available, the analyst performing pairwise or network meta-analysis may be faced with multiple summary statistics for a given endpoint. We present methods for meta-analysing different statistics summarising survival data on the hazard ratio scale. By incorporating all data presentations in a single analysis, we avoid the potential selection bias associated with conducting an analysis for a single statistic only, and the potential difficulties of interpretation, misleading results and loss of available treatment comparisons associated with conducting separate analyses for different summary measures. The methods described also allow use of the most informative statistic available from each study.

## Competing interests

No specific funding was provided for the preparation of this manuscript. However, some of the methods presented were developed as part of an empirical project funded by GlaxoSmithKline.

## Authors' contributions

BW conducted the statistical analysis and drafted the manuscript. NH conceived the study and developed the winBUGS code. DAS contributed to the design of the study and drafting of the manuscript and validated the statistical analysis. All authors reviewed and approved the manuscript.

## Appendix - WinBUGS code

   *A) Fixed effects analysis*

model{

#Define Prior Distributions

      #On tx effect mean

      beta[1] < -0

      for (tt in 2:nTx){

      beta[tt]~dnorm(0,1.0E-6)

      }

      #On individual study baseline effect

      for(ss in 1:nStudies){

      alpha[ss] ~ dnorm(0,1.0E-6)

      }

#Fit data

      #For hazard ratio reporting studies

      for(ii in 1:LnObs ){

      Lmu[ii] < - alpha[Lstudy[ii]]*multi[ii] + beta[Ltx[ii]] - beta[Lbase[ii]]

      Lprec[ii] < - 1/pow(Lse[ii],2)

      Lmean[ii] ~ dnorm(Lmu[ii],Lprec[ii])

      }

      #For binary data reporting studies

      for(ss in 1:BnObs){

      logCumHaz[ss] < - alpha[Bstudy[ss]] + beta[Btx[ss]] - beta[Bbase[ss]]

      cumFail[ss] < - 1-exp(-1*exp(logCumHaz[ss]))

      Br[ss] ~ dbin(cumFail[ss], Bn[ss])

      }

# Calculate HRs

      for (hh in 1:nTx) {

      hr[hh] < -exp(beta[hh])

      }

# Ranking plot

      for (ll in 1:nTx) {

         for (mm in 1:nTx) {

         rk[ll,mm] < - equals(ranked(beta[],mm),beta[ll])

         }

      }

}

# Data

      # Data set descriptors

      list(LnObs = 5, BnObs = 8, nTx = 4, nStudies = 5)

      # Log hazard ratio and log hazard data

      Lstudy[]   Ltx[]   Lbase[]   Lmean[]   Lse[]   multi[]

      1   1   1   0   0.066   1

      1   2   1   0.055   0.063   1

      1   3   1   -0.154   0.070   1

      1   4   1   -0.209   0.072   1

      2   2   1   -0.276   0.203   0

      END

      # Binary data

      Bstudy[]   Btx[]   Bbase[]   Br[]   Bn[]

      3   3   1   1   229

      3   1   1   1   227

      4   2   1   4   374

      4   3   1   3   372

      4   4   1   2   358

      4   1   1   7   361

      5   3   1   1   554

      5   1   1   2   270

      END

# Initial values

      list(alpha = c(-0.50,-0.50,-0.50,-0.50,-0.50), beta = c(NA,-0.5,-0.5,-0.5))

      list(alpha = c(0.50,0.50,0.50,0.50,0.50), beta = c(NA,0.5,0.5,0.5))

   *B)Random effects analysis ****(changes required to incorporate random effect in bold)***

model{

#Define Prior Distributions

      #on random tx effect variance

      **sd~dunif(0,5)**

      **reTau < - 2/pow(sd,2)**

      #On tx effect mean

      beta[1] < -0

      for (tt in 2:nTx){

      beta[tt]~dnorm(0,1.0E-6)

      }

      #On individual study baseline effect

      for(ss in 1:nStudies){

         alpha[ss] ~ dnorm(0,1.0E-6)

      }

#Define random effect

      **for (ss in 1:nStudies){**

         **for(tt in 1:nTx){**

         **re[ss,tt]~dnorm(0,reTau)**

         **}**

      **}**

#Fit data

      #For hazard ratio reporting studies

      for(ii in 1:LnObs ){

      Lmu[ii] < - alpha[Lstudy[ii]]*multi[ii] **+ re[Lstudy[ii],Ltx[ii]] -**

      **re[Lstudy[ii],Lbase[ii]] **+ beta[Ltx[ii]] - beta[Lbase[ii]]

      Lprec[ii] < - 1/pow(Lse[ii],2)

      Lmean[ii] ~ dnorm(Lmu[ii],Lprec[ii])

      }

      #For binary data reporting studies

       for(ss in 1:BnObs){

      logCumHaz[ss] < - alpha[Bstudy[ss]] **+ re[Bstudy[ss],Btx[ss]] -**

      **re[Bstudy[ss],Bbase[ss]] **+ beta[Btx[ss]] - beta[Bbase[ss]]

      cumFail[ss] < - 1-exp(-1*exp(logCumHaz[ss]))

      Br[ss] ~ dbin(cumFail[ss], Bn[ss])

         }

# Calculate HRs

      for (hh in 2:nTx) {

      hr[hh] < -exp(beta[hh])

      }

# Ranking plot

      for (ll in 1:nTx) {

         for (mm in 1:nTx) {

         rk[ll,mm] < - equals(ranked(beta[],mm),beta[ll])

         }

      }

}

# Data as for fixed effects analysis

      ############################

# Initial values

      list(alpha = c(-0.50,-0.50,-0.50,-0.50,-0.50), beta = c(NA,-0.5,-0.5,-0.5),**sd = 0.1**)

      list(alpha = c(0.50,0.50,0.50,0.50,0.50), beta = c(NA,0.5,0.5,0.5),**sd = 1**)

   *C) Additional code required for data reported as median survival times*

   for (ii in 1:medianNObs ){

      medianMu[ii] < - alpha[medianStudy[ii]] + beta[medianTx[ii]] -

      beta[medianBase[ii]]

      prob[ii] < - exp(-median[ii]*exp(medianMu[ii]))

      medianR[ii] ~ dbin(prob[ii],medianN[ii])

      }

## Pre-publication history

The pre-publication history for this paper can be accessed here:

http://www.biomedcentral.com/1471-2288/10/54/prepub
